# Preoperative docetaxel, cisplatin, and 5-fluorouracil for resectable locally advanced esophageal and esophagogastric junctional adenocarcinoma

**DOI:** 10.1007/s10388-024-01050-2

**Published:** 2024-03-12

**Authors:** Toshiharu Hirose, Shun Yamamoto, Yoshitaka Honma, Kazuki Yokoyama, Hidekazu Hirano, Natsuko Okita, Hirokazu Shoji, Satoru Iwasa, Atsuo Takashima, Koshiro Ishiyama, Junya Oguma, Hiroyuki Daiko, Shin Maeda, Ken Kato

**Affiliations:** 1 Department of Head and Neck, Esophageal Medical Oncology, National Cancer Center Hospital, 5-1-1 Tsukiji, Chuo-ku, Tokyo, 104-0045 Japan; 2 Department of Gastrointestinal Medical Oncology, National Cancer Center Hospital, Tokyo, Japan; 3 Department of Esophageal Surgery, National Cancer Center Hospital, Tokyo, Japan; 4 Department of Gastroenterology, Graduate School of Medicine, Yokohama City University, Yokohama, Kanagawa Japan

**Keywords:** Cisplatin, Docetaxel, Esophagogastric junction adenocarcinoma, Fluorouracil, Preoperative chemotherapy

## Abstract

**Background:**

Chemotherapy consisting of 5-fluorouracil, leucovorin, oxaliplatin, and docetaxel is the standard perioperative treatment for resectable esophageal adenocarcinoma and esophagogastric junctional adenocarcinoma (EGJ-AC) in Western countries. Meanwhile, preoperative chemotherapy consisting of docetaxel, cisplatin, and 5-fluorouracil (DCF) has been developed for esophageal squamous cell carcinoma in Japan. However, there are few reports on the safety and efficacy of preoperative DCF for resectable EGJ-AC in the Japanese population.

**Methods:**

Patients with histologically confirmed resectable EGJ-AC who received preoperative DCF (docetaxel 70 mg/m^2^ and cisplatin 70 mg/m^2^ on day 1 and continuous infusion of 5-fluorouracil 750 mg/m^2^/day on days 1–5 every 3 weeks with a maximum of three cycles) between January 2015 and April 2020 were retrospectively evaluated. We assessed the rates of completion of ≥ 2 courses of DCF and R0 resection, histopathological response, progression-free survival (PFS), overall survival (OS), and adverse events.

**Results:**

Thirty-two patients were included. Median follow-up was 28.7 (range, 5.2–70.8) months and median age was 63 (range, 42–80) years. Twenty-one patients (66%) had a performance status of 0. The proportions of clinical stage IIA/IIB/III/IVA/IVB disease were 3%/0%/44%/44%/9%, respectively. The treatment completion rate was 84%. A histopathological response of grade 1a/1b/2/3 was obtained in 58%/26%/13%/3% of cases. Median PFS was 40.7 months (95% confidence interval 11.8-NA). Median OS was not reached (80.8% at 3 years). Grade ≥ 3 adverse events were observed in 63% of cases (neutropenia, 44%; febrile neutropenia, 13%). No treatment-related deaths occurred.

**Conclusions:**

Preoperative DCF for resectable EGJ-AC was well tolerated and has promising efficacy.

## Introduction

Esophageal cancer is the seventh most common cancer and the sixth leading cause of cancer deaths worldwide [1]. There are two main histological subtypes of esophageal cancer, esophageal squamous cell carcinoma (ESCC) and esophageal adenocarcinoma (EAC). The majority of ESCCs are located in the mid-esophagus whereas most EACs are located in the lower esophagus or esophagogastric junction (EGJ). ESCC is the predominant histological subtype in Japan, accounting for about 86% of all cases, followed in frequency by EAC, which accounts for about 7% of all cases [2]. For the past three decades, the frequency of EAC and EGJ cancers originating from the gastric cardia and distal esophagus has increased dramatically. EAC is the most common histological type of esophageal cancer in Western countries [3].

Unlike ESCC, the molecular profile of EAC is similar to that of gastric cancer [4]. Therefore, development of treatment for EAC has proceeded in a similar fashion as that for gastric cancer. Surgical resection is the curative modality for patients without distant disease, but surgical resection alone is associated with poor clinical outcomes and there have been reports on use of multimodal treatments, such as preoperative chemoradiotherapy or perioperative chemotherapy [5, 6]. However, the optimal perioperative treatment has not yet been established for patients with esophagogastric junctional adenocarcinoma (EGJ-AC).

In Japan, patients with EAC undergo upfront surgery followed by postoperative chemotherapy similar to that in patients with gastric cancer. The JACCRO GC-07 trial found that the 3-year overall survival (OS) rate in patients with advanced gastric cancer was significantly better after postoperative docetaxel plus S-1 therapy than after S-1 alone (65.9 vs 49.5%, hazard ratio 0.63, 95% confidence interval [CI] 0.40–0.99]) [7]. Based on this results, postoperative docetaxel plus S-1 therapy has been used in patients with EGJ-AC.

In Western countries, 5-fluorouracil, leucovorin, oxaliplatin, and docetaxel (FLOT) has been the standard perioperative treatment for patients with resectable gastric AC or EGJ-AC [8]. The FLOT4 trial compared perioperative capecitabine, cisplatin, and epirubicin with perioperative FLOT for gastric and EGJ tumors and found that median OS was significantly longer in patients who received FLOT than in those who received capecitabine, cisplatin, and epirubicin (50 months vs 35 months) [8]. The CROSS regimen has also become one of the standard treatments for locally advanced EAC and EGJ-AC. The long-term results of the CROSS trial have been reported, including a median OS of 43.2 months (95% CI 24.9–61.4) in patients who received this regimen (carboplatin and paclitaxel, 41.4 Gy) [6]. The survival rate was 54% (95% CI 47–64) at 3 years and 36% (95% CI 29–45) at 10 years and the pathological complete response rate was 23%. However, patients with ESCC were also included in that study, and subgroup analysis by histological subtype showed a trend toward worse OS in those with EAC than in those with ESCC.

The Korean PRODIGY trial compared surgery plus postoperative S-1 therapy with preoperative docetaxel, oxaliplatin, and S-1 (DOS) therapy plus surgery for resectable gastric cancer and EGJ-AC. In that study, the 3-year PFS rate was 66.3% in the group that received DOS therapy and 60.2% in the group that received surgery plus postoperative S-1 therapy, indicating a favorable trend for the preoperative group [9]. The longer term results of this trial have since been reported and included a significantly higher 5-year OS rate in the group that received preoperative DOS (66.8 vs 63.0%) [10]. In Japan, where only gastric cancer has been treated, the efficacy of DOS therapy has been reported with a promising pathological complete response rate (24%) [11]. Although such promising regimens of FLOT and DOS have been reported, there are currently few reports on safety and efficacy in Japanese patients with EGJ-AC. There is no established management that allows for safe administration.

Preoperative chemotherapy with docetaxel, cisplatin, and 5-fluorouracil (DCF) was developed for patients with resectable ESCC [12] and found to be significantly more effective than cisplatin and 5-fluorouracil in the JCOG1109 trial [13]. Compared with conventional cisplatin and 5-fluorouracil therapy, DCF has demonstrated a higher response rate and longer postoperative progression-free survival (PFS) in patients with ESCC. Based on these results, DCF therapy has become the standard of treatment for resectable ESCC. DCF therapy is a manageable regimen in the Japanese population, including adverse events.

As in esophageal cancer, preoperative chemotherapy plus surgery may be an appropriate treatment strategy in patients with EAC or EGJ-AC. Furthermore, powerful chemotherapy is necessary to prevent recurrence, considering the biology of these tumors. There are few reports on the safety and efficacy of preoperative triple therapy for patients with resectable EGJ-AC in Japan. Therefore, in this study, we evaluated the efficacy and safety of preoperative DCF plus esophagectomy and mediastinal lymph node dissection for resectable locally advanced EAC and EGJ-AC, as is performed for ESCC.

## Patients and methods

### Patient eligibility

We retrospectively reviewed the medical records of patients with EGJ-AC who received preoperative DCF before planned surgery at our hospital between January 2015 and April 2020. The standard surgical technique for EGJ-AC is subtotal esophagectomy with 2- or 3-field lymph node dissection, performed via an open thoracotomy or thoracoscopic approach. All patients had histologically confirmed EGJ-AC with clinically diagnosed resectable locally advanced disease (cT1N1-3M0, cT2-3N0-3M0, or cT1-3N0-3M1). M1 was limited to supraclavicular lymph node metastasis. Barrett's esophagus was not included.

The study was approved by the Institutional Review Board of the National Cancer Center Hospital (approval number: 2020-287). Informed consent was obtained from all patients via the opt-out route.

### Treatment

Preoperative chemotherapy consisted of two or three courses of docetaxel (70 mg/m^2^, day 1), cisplatin (70 mg/m^2^, day 1), and 5-fluorouracil (750 mg/m^2^, days 1–5) administered every 3 weeks. All patients who were able to swallow received prophylactic oral ciprofloxacin (600 mg/day, days 5–15).

### Evaluation of efficacy and safety

Efficacy was assessed based on the rates of treatment completion, histopathological response, PFS, and OS. The treatment completion rate was defined as the proportion of patients who completed ≥ 2 courses of DCF and underwent R0 resection. Histopathological response was evaluated according to the Japanese Classification of Esophageal Cancer, 11th Edition (grade 0, no area of degeneration; grade 1a, viable tumor cells ≥ 2/3; grade 1b, 2/3 > viable tumor cells ≥ 1/3; grade 2, viable tumor cells < 1/3; grade 3, no viable tumor). PFS was defined as the interval between initiation of treatment and disease progression, incomplete (R1/R2) resection, relapse, or death from any cause, whichever came first. OS was defined as the interval between initiation of treatment and death from any cause.

Clinical staging was determined according to the 8th edition of the Union for International Cancer Control TNM classification by a multidisciplinary team after discussion based on endoscopy, thin-slice computed tomography, and positron emission tomography findings if needed. Adverse events were evaluated according to the Common Terminology Criteria for Adverse Events version 5.02.

### Statistical analysis

Survival was estimated using the Kaplan–Meier method. All statistical analyses were performed in EZR (Easy R), which is a modified version of R Commander designed to add frequently used statistical functions in biostatistics.

## Results

### Patient characteristics and treatment

Thirty-two patients with histologically confirmed resectable EGJ-AC were treated at our hospital between January 2015 and April 2020. Median age was 63 (range, 42–80) years and 31 patients (97%) were male. The characteristics of the patients are shown in Table 1. Siewert type 2 was the most common location. HER2 status was positive in only 6% of patients. All patients were able to take orally.

**Table 1:** Patient demographic and clinical characteristics

Characteristic		N = 32
Sex	Male	31 (97)
	Female	1 (3)
Age, years, median (range)		63 (42–80)
ECOG PS	0	21 (66)
	1	11 (34)
Siewert type	1	9 (28)
	2	21 (66)
	3	2 (6)
Barrett ‘s carcinoma	Yes	0 (0)
	No	32 (100)
Clinical T stage	cT1b	1 (3)
	cT2	3 (9)
	cT3	28 (88)
Clinical N stage	cN0	4 (12)
	cN1	13 (41)
	cN2	13 (41)
	cN3	2 (6)
Clinical M stage	cM0	29 (91)
	cM1	3 (9)
Clinical stage	IIA	1 (3)
	IIB	0 (0)
	III	14 (44)
	IVA	14 (44)
	IVB	3 (9)
Histology	Well differentiated	5 (16)
	Moderate differentiated	12 (37)
	Poorly differentiated	10 (31)
	Signet ring cell	5 (16)
HER2 status	0	14 (44)
	1+	5 (16)
	2+ DISH-negative	4 (12)
	2+ DISH-positive	2 (6)
	3+	0 (0)
	Unknown	7 (22)
NLR, median (range)		0.30 (0.11–1.69)
PNI, median (range)		48.7 (35.1–56.3)

Data are shown as the number (percentage) unless otherwise stated. *DISH* dual in situ hybridization; *ECOG* Eastern Cooperative Oncology Group; *HER2* human epidermal growth factor receptor 2; *NLR* Neutrophil-Lymphocyte Ratio; *PNI* Prognostic Nutritional Index; *PS* performance status

**Fig. 1:**
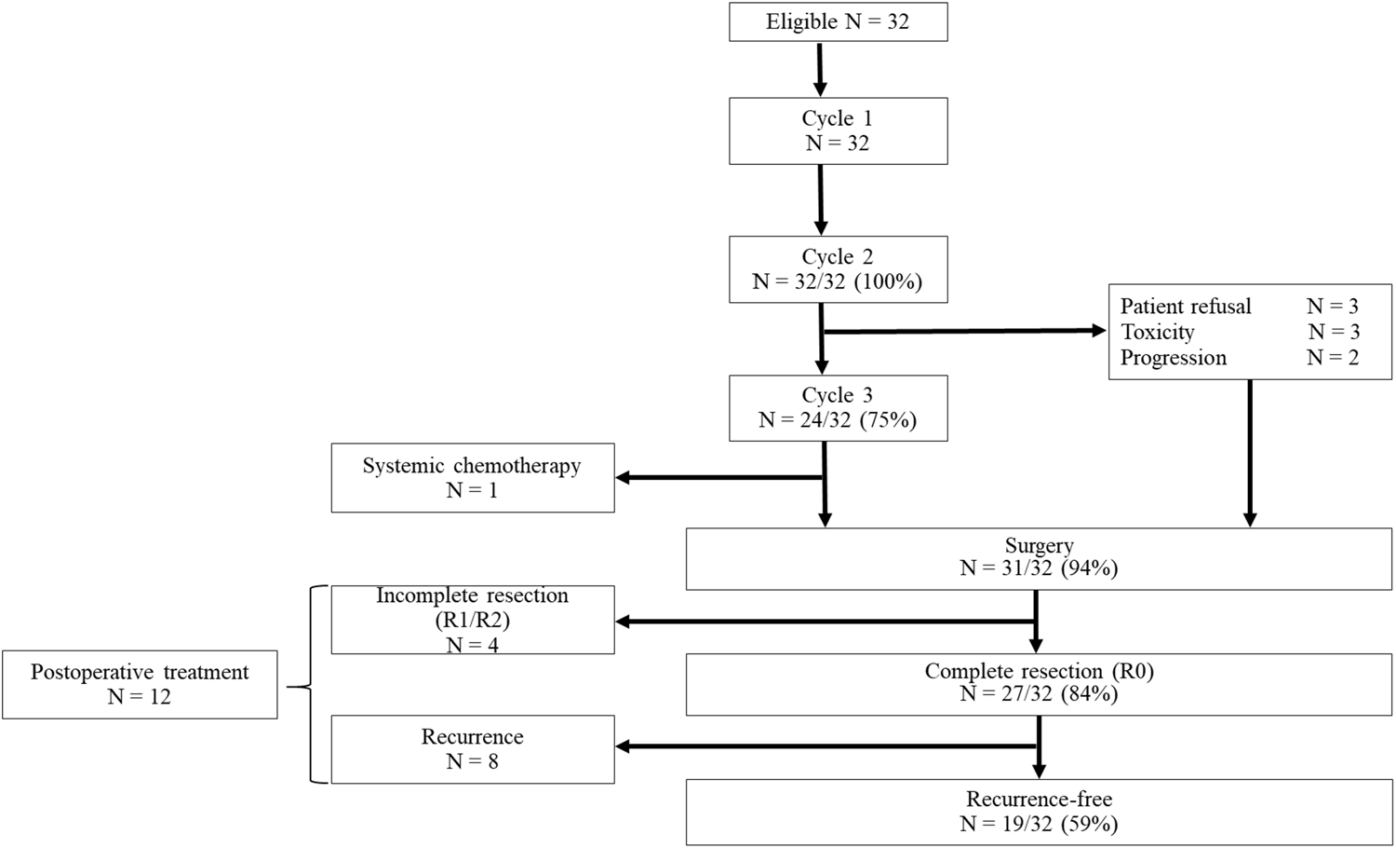
Flow chart showing the surgery and chemotherapy received by the study participants

Eight of the 32 patients ceased preoperative therapy in the second cycle because of toxicity (grade 3 anorexia, n = 2; grade 4 neutropenia, n = 1), patient refusal to continue (n = 3), or progression of disease (n = 2). Finally, 24 of the 32 patients (75%) received three cycles of DCF. One patient was found to have bone metastases before surgery and received systemic palliative chemotherapy. Thirty-one of the 32 patients (94%) underwent surgery (Fig. 1). Twenty of 32 patients (63%) required at least one cycle of dose modification of any drug. The median relative dose intensity (actual dose/planned dose) was 0.93 (range, 0.2–1.0) for docetaxel, 0.93 (range, 0.6–1.0) for cisplatin, and 0.86 (range, 0.6–1.0) for 5-fluorouracil 5-FU, respectively.

### Efficacy

Complete (R0) resection was achieved in 27 of the 32 patients who received DCF therapy (84%). R1 resection and R2 resection were achieved in 2 patients each. All twenty-seven patients who achieved R0 resection received two or more cycles of DCF, giving a treatment completion rate of 84%. Thirteen patients (42%) achieved a histopathological response of grade 1b or better. A pathological complete response was observed in 1 patient (3%) (Table 2).

**Table 2:** Surgical and pathological outcomes

Outcome		Number of patients (%) (N = 31)
Lymph node dissection	3-field	28 (90)
	2- field	3 (10)
Residual tumor	R0	27 (87)
	R1	2 (6)
	R2	2 (6)
Histological therapeutic effect	Grade 1a	18 (58)
	Grade 1b	8 (26)
	Grade 2	4 (13)
	Grade 3	1 (3)
Pathological T stage	≤ ypT1	6 (19)
	ypT2	6 (19)
	ypT3	18 (56)
	ypT4	1 (3)
	Not applicable	1 (3)
Pathological N stage	ypN0	8 (25)
	ypN1	10 (31)
	ypN2	7 (22)
	ypN3	6 (19)
	Not applicable	1 (3)
Postoperative complications	Recurrent nerve palsy	8 (26)
	Anastomotic leakage	7 (23)
	Pneumonia	5 (16)
	Acute circulatory failure	3 (10)
	Lymphorrhea	1 (3)
	Postoperative mortality	0 (0)

**Fig. 2:**
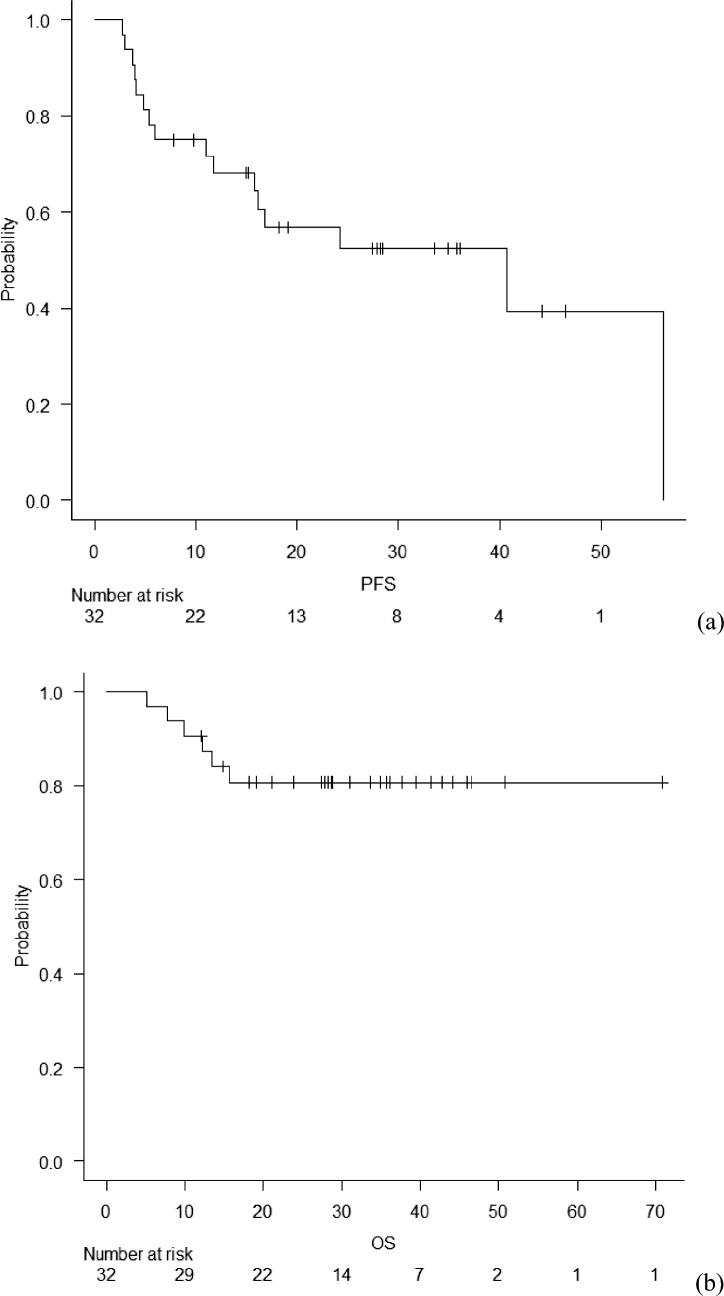
Kaplan–Meier estimates of **a** PFS and **b** OS. Median PFS was 40.7 (range, 11.8-NA) months. Median OS was not reached. The estimated 3-year OS rate was 80.8%. *NA* not achieved; *OS* overall survival; *PFS* progression-free survival

Median follow-up duration was 28.7 (range, 5.2–70.8) months and median PFS was 40.7 (range, 11.8-NA) months (Fig. 2a). Median OS was not reached, and the 3-year OS rate was 80.8% (Fig. 2b).

Nineteen of the 32 patients were alive without recurrence at the time of data cut-off. Eight of 27 patients who underwent complete resection had recurrence. The most common recurrence site was distant metastasis (adrenal gland, lung, liver, peritoneal, lymph node, or bone), documented in 7 cases. Thirteen patients received treatment for recurrent or residual disease. As mentioned above, 1 patient received palliative chemotherapy without surgery after bone metastases were found preoperatively. Chemotherapy was administered in 4 patients after resection was found to be R1 or R2 postoperatively and in 8 when distant metastatic recurrence was found. No adjuvant chemotherapy was given when R0 resection was achieved. One of the 12 patients with residual or recurrent disease underwent salvage surgery, 10 received palliative chemotherapy, and 1 who was in poor overall condition received best supportive care. Platinum-based chemotherapy was the most common chemotherapy regimen, administered in 8 patients. Immune checkpoint inhibitors were used in 4 patients (Fig. 1, Table 3).

**Table 3:** Details of recurrence and subsequent treatment

Status	Number of patients	
Recurrence	Locoregional	1
	Distant	7
	Total	8
Metastatic site in distant relapse	Adrenal gland	2
	Lung	1
	Liver	1
	Peritoneum	1
	Para-Ao LN	1
	Bone	1
	Total	7
Postoperative treatment	Palliative chemotherapy	10
	Surgery	1
	Best supportive care	1
	Total	12
Type of chemotherapy	CDDP-based	3
	L-OHP-based	5
	CPT-11-based	3
	PTX-based	3
	Immune checkpoint inhibitors	4
	Total	10

*CDDP* cisplatin; *CPT-11* irinotecan; *L-OHP* oxaliplatin; *Para-Ao L*, paraaortic lymph nodes; *PTX* paclitaxel

### Toxicity

The toxicity profile of DCF is shown in Table 4. Hematological toxicity was the most common adverse event. Fourteen patients (43%) had grade 3 or 4 neutropenia. Febrile neutropenia was observed in 4 patients (12%). Anorexia and nausea were the most common non-hematological toxicities, occurring in 27 patients (84%) and 28 patients (88%), respectively, and specifically in 3 (9%) patients each among those who had grade 3–4. There were no treatment-related deaths.

**Table 4:** Adverse events during preoperative chemotherapy

Adverse event	Grade 1	Grade 2	Grade 3	Grade 4	Grade ≥ 3	All grades
Hematological						
Leukopenia	3 (9)	9 (28)	5 (16)	2 (6)	7 (22)	19 (59)
Neutropenia	6 (19)	4 (12)	10 (31)	4 (12)	14 (43)	24 (75)
Anemia	26 (81)	3 (9)	0 (0)	0 (0)	0 (0)	29 (91)
Thrombocytopenia	14 (44)	0 (0)	0 (0)	0 (0)	0 (0)	14 (44)
Non-hematological						
Anorexia	14 (44)	10 (31)	3 (9)	0 (0)	3 (9)	27 (84)
Malaise	10 (31)	3 (9)	0 (0)	0 (0)	0 (0)	13 (40)
Nausea	18 (56)	7 (22)	3 (9)	0 (0)	3 (9)	28 (88)
Vomiting	2 (6)	1 (3)	1 (3)	0 (0)	1 (3)	4 (12)
Diarrhea	8 (25)	4 (12)	2 (6)	0 (0)	2 (6)	14 (44)
Mucositis	7 (22)	7 (22)	2 (6)	0 (0)	2 (6)	16 (50)
Febrile neutropenia	–	–	4 (12)	0 (0)	4 (12)	4 (12)

Data are shown as the number (percentage)

Postoperative complications are shown in Table 2. Recurrent nerve palsy was the most common complication, occurring in 8 of the 31 patients (26%), followed by anastomotic leak in 7 patients (23%). Postoperative complications were not significantly different from those already known.

## Discussion

All patients in this study received at least two cycles of chemotherapy, and curative resection was achieved in 27 patients, giving a treatment completion rate of 84%. In terms of efficacy, median PFS was 40.7 (range, 11.8-NA) months. Three-year OS was 80.8% and median OS was not reached. Although the histological complete response rate was low at 3%, long-term efficacy was more promising than that with the FLOT regimen. In addition, adverse events were well tolerated.

A previous multicenter Phase II trial in Canada demonstrated the efficacy of perioperative DCF for locally advanced esophageal or gastric adenocarcinoma [14]. In that study, a total of 43 patients had adenocarcinoma, 11 in the esophagus, 25 in the gastroesophageal junction, and 7 in the stomach. Complete resection was achieved in 41 patients (95.3%) and a pathological complete response in 4 (9%). The 3-year OS rate was 60%. However, this DCF regimen was based on a higher dose of docetaxel and cisplatin (75 mg/m^2^ for both), which is different from the DCF regimen in our study.

The pathological complete response rate was lower in our study than in the Canadian, FLOT, and CROSS trials, but our PFS and OS rates were better. Recurrence of distant metastasis was the main site of recurrence in our study (7/26, 26.9%), with only 1 local recurrence (1/26, 3.8%). This local recurrence rate was lower than that reported in the earlier trials. This discrepancy between short-term efficacy and long-term efficacy might reflect the extent of surgical lymph node dissection. In our study, 3-field dissection was performed in 25 patients (81%), which is a higher rate than in the previous studies. In previous reports, lymph node metastasis in the upper mediastinum and middle or lower mediastinum was frequent in both Siewert type I and II disease [15]. In those studies, 3-field dissection was rarely performed, so this might have contributed to our lower local recurrence and better survival outcomes. Surgery with 3-field dissection after intensive preoperative chemotherapy might provide better local control.

Patients in our study received preoperative chemotherapy, but did not receive postoperative chemotherapy. Only about half of the patients in the previous studies received postoperative chemotherapy because of the invasive nature of esophageal surgery. This suggests that our treatment improved survival by introducing more appropriate postoperative chemotherapy, which would be expected to reduce the rate of distant metastatic recurrence.

In terms of toxicity, 19 patients (59%) experienced grade 3 or 4 adverse events during treatment with DCF. Consistent with a previous report, the most common grade 3 and 4 adverse events were neutropenia (43%) and leucopenia (22%) [12]. Febrile neutropenia was observed in only 4 patients (12%). There were no unexpected postoperative complications in this study, indicating a similar safety profile to that reported previously [14]. Preoperative DCF was recently reported to improve survival in patients with locally advanced ESCC [13] and is now a standard therapy. In the JCOG1109 study, neutropenia of grade ≥ 3 was reported in 85% of patients and leukopenia in 64%, with febrile neutropenia in 16%. Adverse events can be managed with appropriate supportive care, such as prophylactic antibiotic therapy. Prophylactic pegfilgrastim on day 3 was reported to reduce the rate of febrile neutropenia from 20 to 6% in high-risk patients, including the elderly [16], suggesting that this regimen will be more manageable in the future. On the other hand, grade 3 or more neutropenia was reported in 51% of patients in the FLOT therapy [8]. Safety reports are scarce in Japan, and the appropriate management of neutropenia is not yet known. In this regard, DCF therapy has the advantage that it can be administered safely while maintaining an appropriate dose intensity.

This study had several limitations. First, it was designed for resectable EGJ-AC, and patients with clinical T4 disease were not included. Only 1 patient (3%) was found to have ypT4 disease on postoperative pathology. Patients with ypT4 disease accounted for about 10% of all cases in previous studies, so our single case may have contributed to the favorable results in the present study. Second, this study had a single-center retrospective design and the number of patients analyzed was small. Further validation is needed in a larger sample size. Finally, our study included only patients with preserved cardiac and renal function who could tolerate the nephrotoxicity and high-volume hydration required by cisplatin. Patients were included with favorable renal and cardiac function compared to patients who had received oxaliplatin-based therapy, and these patients may have contributed to the prolonged prognosis. Nevertheless, we believe that this study is important because it is one of few studies to have examined preoperative triplet chemotherapy in patients with EGJ-AC to date.

In summary, all of our patients were treated for at least two cycles and curative resection was achieved in 27 patients (84%). The treatment completion rate was 84%. Preoperative DCF chemotherapy for resectable EGJ-AC was well tolerated. Further investigation is needed to evaluate the long-term efficacy of this treatment strategy. 

## Data Availability

The data that support the findings of this study are available on request from the corresponding author, KK. The data are not publicly available due to their containing information that could compromise the privacy of research participants.

## References

[1] World Health Organization. GLOBOCAN 2020 estimated cancer incidence, mortality and prevalence worldwide. http://globocan.iarc.fr/. Accessed 22 Mar 2022

[2] Kitagawa Y, Ishihara R, Ishikawa H (2023). Esophageal cancer practice guidelines 2022 edited by the Japan esophageal society: part 2. Esophagus..

[3] Thrift AP (2016). The epidemic of oesophageal carcinoma: Where are we now?. Cancer Epidemiol.

[4] Dulak AM, Stojanov P, Peng S (2013). Exome and whole-genome sequencing of esophageal adenocarcinoma identifies recurrent driver events and mutational complexity. Nat Genet.

[5] Ychou M, Boige V, Pignon J (2011). Perioperative chemotherapy compared with surgery alone for resectable gastroesophageal adenocarcinoma: an FNCLCC and FFCD multicenter phase III trial. J Clin Oncol.

[6] Shapiro J, Lanschot JJB, Hulshof MCCM (2015). Neoadjuvant chemoradiotherapy plus surgery versus surgery alone for oesophageal or junctional cancer (CROSS): long-term results of a randomised controlled trial. Lancet Oncol.

[7] Yoshida K, Kodera Y, Kochi M (2019). Addition of docetaxel to oral fluoropyrimidine improves efficacy in patients with stage III gastric cancer: interim analysis of JACCRO GC-07, a randomized controlled trial. J Clin Oncol.

[8] AI-BartranHomannPauligk SENC (2019). Perioperative chemotherapy with fluorouracil plus leucovorin, oxaliplatin, and docetaxel versus fluorouracil or capecitabine plus cisplatin and epirubicin for locally advanced, resectable gastric or gastro-oesophageal junction adenocarcinoma (FLOT4): a randomised, phase 2/3 trial. Lancet..

[9] Kang YK, Yook JH, Park YK (2021). PRODIGY: a phase iii study of neoadjuvant docetaxel, oxaliplatin, and S-1 plus surgery and adjuvant S-1 versus surgery and adjuvant S-1 for resectable advanced gastric cancer. J Clin Oncol.

[10] Kang YK, Kim HD, Yook JH (2023). Neoadjuvant docetaxel, oxaliplatin, and s-1 plus surgery and adjuvant s-1 for resectable advanced gastric cancer: final survival outcomes of the randomized phase 3 PRODIGY trial. J Clin Oncol.

[11] Kurokawa Y, Doki Y, Kitabayashi R (2024). Short-term outcomes of preoperative chemotherapy with docetaxel, oxaliplatin, and S-1 for gastric cancer with extensive lymph node metastasis (JCOG1704). Gastric Cancer.

[12] Nakamura K, Kato K, Igaki H (2013). Three-arm phase III trial comparing cisplatin plus 5-FU (CF) versus docetaxel, cisplatin plus 5-FU (DCF) versus radiotherapy with CF (CF-RT) as preoperative therapy for locally advanced esophageal cancer (JCOG1109, NExT study). Jpn J Clin Oncol.

[13] Kato K, Ito Y, Daiko H (2022). A randomized controlled phase III trial comparing two chemotherapy regimen and chemoradiotherapy regimen as neoadjuvant treatment for locally advanced esophageal cancer, JCOG1109 NExT study. J Clin Oncol.

[14] Ferri LE, Ades S, Alcindor T (2012). Perioperative docetaxel, cisplatin, and 5-fluorouracil (DCF) for locally advanced esophageal and gastric adenocarcinoma: a multicenter phase II trial. Ann Oncol.

[15] Sakaki A, Kanamori J, Ishiyama K (2020). Distribution of lymph node metastases in locally advanced adenocarcinomas of the esophagogastric junction (cT2-4): comparison between Siewert type I and selected Siewert type II tumors. Langenbecks Arch Surg.

[16] Ikeda G, Ohara A, Itoyma M (2022). Efficacy of prophylactic pegfilgrastim on day three of preoperative DCF chemotherapy in elderly patients with resectable esophageal cancer. J Clin Oncol.

